# Official Report of the Results of Koch’s Treatment

**Published:** 1891-05

**Authors:** 


### OFFICIAL REPORT OF THE RESULTS OF KOCH'S TREATMENT.

The official report on the results of Koch's treatment in Prussian hospitals has been published in *Klinisches Jahrbuch*, in which it is stated that out of a total number of 1061 patients treated for tuberculosis of internal organs 13 were cured, 194 improved, 171 substantially improved, 586 unimproved, and 46

died. In external tuberculosis (lupus, scrofula, tubercular joints, etc.) 708 cases were treated; of these 15 were cured, 237 improved, 148 substantially improved, 298 unimproved, and 9 died.

We must confess that this report, which seems to have been made in all candor, is not at all encouraging. Under our old methods of treatment we can obtain some cures and many improvements. The new method does not seem to have accomplished very much more.

